# Potato tuber degradation is regulated by carbohydrate metabolism: Results of transcriptomic analysis

**DOI:** 10.1002/pld3.379

**Published:** 2022-01-14

**Authors:** Liguo Jia, Kai Hao, Qiqige Suyala, Yonglin Qin, Jing Yu, Kun Liu, Mingshou Fan

**Affiliations:** ^1^ College of Agronomy Inner Mongolia Agricultural University Hohhot China; ^2^ College of Grassland and resource environment Inner Mongolia Agricultural University Hohhot China

**Keywords:** carbohydrate, potato, RNA‐sequencing, sink, source, tuber degradation

## Abstract

Tuber number is an essential factor determining yield and commodity in potato production. The initiation number has long been considered the sole determinant of the final total tuber number. In this study, we observed that tuber numbers at harvest were lower than at the tuber bulking stage; some formed tubers that were smaller than 3 cm degraded during development. Carbohydrate metabolism plays a crucial role in tuber degradation by coordinating the source–sink relationship. The contents of starch and sucrose, and the C:N ratio, are dramatically reduced in degradating tubers. Transcriptomic study showed that “carbohydrate metabolic processes” are Gene Ontology (GO) terms associated with tuber degradation. A polysaccharide degradation‐related gene, LOC102601831, and a sugar transport gene, LOC102587850 (SWEET6a), are dramatically up‐regulated in degradating tubers according to transcriptomic analysis, as validated by qRT‐PCT. The terms “peptidase inhibitor activity” and “hydrolase activity” refer to the changes in molecular functions that degradating tubers exhibit. Nitrogen supplementation during potato development alleviates tuber degradation to a certain degree. This study provides novel insight into potato tuber development and possible management strategies for improving potato cultivation.

## 1 INTRODUCTION

Potato (*Solanum tuberosum L*.) is one of the most important food crops globally, with the planting area and total yield reaching 17.34 million hectares and 104.67 megatons, respectively, in 2019 (FAO: http://www.fao.org/faostat/en/#data/QCL). Potato plays a particularly important role in food security in developing regions, as a food that is rich in carbohydrates and a wide variety of vitamins but with relatively low calorie content (Romero et al., 2017). Therefore, further increasing potato yield and understanding the physiological mechanisms are crucial given the world's ever‐growing population.

Unlike other staple crops, such as rice, maize, and wheat, the potato organs harvested are tubers that grow underground. Understanding of the formation process—tuberisation—has advanced considerably in recent decades. The tuber initiates from a hooked, unswollen stolon in the subapical region (Xu et al., 1998). Several leaf‐derived signals are involved in tuberization, including FT (FLOWERING LOCUS T) protein (Abelenda et al., 2014; Navarro et al., 2011), BEL (BELLRINGER‐1 like), mRNA Bel5 (Banerjee et al., 2006; Cho et al., 2015), miR156, and miRNA 172 (Bhogale et al., 2014; Martin et al., 2009). Environmental factors, such as photoperiod, light quality, temperature, water, and nutrient supply, influence tuber development (Suyala et al., 2017; Zierer et al., 2021).

Relative source and sink strength also play an essential role in regulating tuber formation. From the onset of tuber development, the swollen part of the stolon, as strong sink tissue, imports assimilates from source organs. Carbohydrates, particularly sucrose, are considered to be the primary means by which assimilates are transported from source to sink organ. During the early 1980s, sucrose was reported to induce tuber initiation without the addition of growth‐regulating substances to the culture medium (Garner & Jennet, 1989). Evidence has also revealed that sucrose is an important material in potato tuber development; indeed, the transportation of sucrose in phloem links the source and sink.

The sucrose synthesized in mesophyll cells first diffuses symplasmically to the phloem parenchyma–companion cell border (Zierer et al., 2021). A sucrose efflux carrier, SUGARS WILL EVENTUALLY BE EXPORTED TRANSPORTER (SWEET), anchored at the membrane exports sucrose from parenchyma cells into the sieve element‐companion cell complex (Braun, 2012; Chen et al., 2012). The sucrose is actively transported by the sucrose/H^+^ symporter, SUCROSE TRANSPORTER (StSUT1 and StSUT4), into companion cells (Riesmeier, 1993; Riesmeier et al., 1994). Soluble carbohydrate concentrations more than 20 times higher accumulated in the leaves of the StSUT4‐RNAi plants, leading to modified sucrose levels in sink organs (Abelenda et al., 2019; Chincinska et al., 2008). The sucrose concentration gradient built up in this way allows long‐distance transport via mass flow through sieve elements to the sink organ.

In sink tissue, StSUT1, a protein expressed in the tubers' sieve elements, contributes to sucrose retrieval from apoplasm (Kühn et al., 2003). StSUT2 and StSUT4 transport sucrose with low affinity in sink tubers (Chincinska et al., 2008). Simultaneous with the first visible signs of tubers, the sucrose unloading pathway switches from apoplastic to symplastic (Viola et al., 2001). Moreover, the activity of invertases decreases dramatically in the subapical region of swelling stolons, while sucrose synthase (SuSy) plays a major role in sucrose cleavage with tuberization being a determinant of tuber sink strength (Zrenner et al., 1995).

Sucrose, not only as a metabolite but also as a signal component, regulates the sink–source relationship and tuber development. Sucrose, together with trehalose 6‐phosphate (Tre6P), forms the Suc–Tre6P nexus, linking growth and development to plant carbon (C) status. As a signal, Tre6P regulates assimilate partitioning during the day and transitory starch breakdown at night in source leaves, thereby maintaining optimal sucrose levels (Figueroa & Lunn, 2016; O'Hara et al., 2013). In sink tuber organs, Tre6P influences sucrose remobilization, and OtsA‐overexpressing lines with elevated Tre6P exhibit changes in starch content (Debast et al., 2011; Kolbe et al., 2005). The level of Tre6P is also feedback‐regulated by sucrose via the activation of Tre6P synthase (TPS), inhibition of Tre6P phosphatase (TPP), or both (Baena‐González & Lunn, 2020).

In addition to C, nitrogen (N) and the C:N ratio play crucial roles in the source–sink relationship and tuber development. Suitable N supply is beneficial for chlorophyll biogenesis and photosynthesis, and thus for source strengthening. However, continuous N supply or large amounts of available N delay tuber formation (Krauss & Marschner, 1982; Sarkar & Naik, 1998). The first report concerning the C:N ratio and tuberization dates back almost a century and found that high C:N promotes tuber formation (Wellensiek, 1929). This conclusion was verified by a recent study (Zheng et al., 2018). In fact, regulation of tuberisation by the C:N ratio maybe partially explained by source–sink theory.

Carbohydrate metabolism and source–sink balance thus play important roles in tuber development. It is generally accepted that the formed tuber will enlarge gradually and that the final size will vary based on the formation timing and surrounding conditions. However, we found that not all formed tubers grew continuously; some disappeared before harvest. Whether degradation occurs as a result of sink competition and the precise mechanism underlying this putative phenomenon are unknown. To date, no report has been published on this issue, so we investigated it in the present study.

## 2 MATERIALS AND METHODS

### 2.1 Plant materials and growth conditions

The experiments were conducted on a farm located in Chayouzhong County, Inner Mongolia Autonomous Region, China (41°27′N, 112°63′E) from 2017 to 2019. The average photosynthetically active radiation (PAR) amount per year was approximately 1.83 × 10^6^ kJ/m^2^, and the mean temperature was 14.2°C (range: 8.2–28.5°C) during the potato‐growing season (May to September). In 2017, field managers implemented local potato production practices. The rainfall was 229 and 266 mm during the potato growth periods in 2018 and 2019, respectively. The soil was a sandy loam (chestnut; Chinese classification), and its sand, silt, and clay contents were approximately 50%, 30%, and 14%, respectively. The gravel content (>2 mm in diameter) was 6%. The soil properties of the experimental sites are as follows: 16.5 g/kg organic matter, 1.02 g/kg total N, 9.52 mg/kg Olsen‐P, and 124 mg/kg exchangeable K in 2018 and 18.1 g/kg organic matter, 1.19 g/kg total N, 15.01 mg/kg Olsen‐P, and 133 mg/kg exchangeable K in 2019(in a 0–40 cm soil layer). The pH values were 7.9 and 7.7 in 2018 and 2019, respectively. The preceding crop in the experimental fields was spring wheat.

The popular local potato (*S. tuberosum L*.) cultivar, Favorita, was used, and virus‐free seed potato was provided for the trials. Two more cultivars, Kexin No. 1 and Macon, were used for investigation in 2017. Each year, 180 kg/ha P_2_O_5_ as single superphosphate and 300 kg/ha K_2_O as K_2_SO_4_ were applied as basal dressing. Two treatments, control (75 kg/ha N as basal dressing, no top‐dressing) and Sup N (75 kg/ha N as basal dressing and 225 kg/ha N as top‐dressing), were implemented in both years. In the Sup N treatment, a 45‐kg/ha aliquot of N was applied as top‐dressing by drip fertigation, five times at 10‐day intervals beginning 18 days after emergence (DAE). The chemical fertilizer N was applied as urea in the trials. A complete randomized block design was used with three replicates. Each plot covered 300 m^2^, with a 90‐cm distance between rows and a 20‐cm distance between seedlings within each row. Weeds, insects, and diseases were controlled in accordance with standard local prevention practices and care recommendations.

### 2.2 Tuber sampling and grading

The tuber numbers were recorded for potato cultivar Kexin No. 1, Favorita, and Macon in 2017.The sampling dates were 20 July (tuber bulking stage) and 20 August (harvest stage). Ten potato plants in a row were randomly selected for sampling and those with a diameter over 0.5 cm were included in the calculation of the total number of tubers.

Tubers were counted in 2018 and 2019. In total, 30 potato plants (10 seedlings per replicate plot) were randomly selected and sampled at 15, 25, 35, 45, 55, and 65 DAE for each treatment. Tubers and tuber initials (<0.5 cm in diameter) were added to obtain the total tuber number. Tubers were classified according to diameter as follows: <0.5, 0.5–1, 1–3, 3–5, and >5 cm. The number of seedlings showing tuber degradation was recorded. Ten seedlings with degradating tuber were selected randomly in each treatment at 45, 55, and 65 DAE, in which the degradating tuber and total tuber numbers were recorded for each seedling. The ratio of degradating tuber and total tuber numbers was regarded as “percentage of degradating tubers.”

### 2.3 Leaf area and tuber dry matter

Leaf area was measured using a leaf area meter (Model LI‐3100; LI‐COR, Inc., Lincoln, NE, USA), and tuber dry weight was measured using the oven‐drying method at 45, 55, and 65 DAE. In total, 10 normal potato seedlings and 10 with degradating tubers were randomly selected. The tubers were separated from the plants, dried for 30 min at 105°C, and kept at 80°C until they reached a constant weight; dry weights were then recorded. The leaf area ratios (m^2^) and tuber dry weights (g) were calculated from the same seedlings.

### 2.4 Histology

Normal and degradating tubers from the same seedlings were fixed in FAA solution and then embedded in paraffin (Sigma‐Aldrich, St Louis, MO, USA); 7‐μm sections were stained with 0.05% (w/v) toluidine blue (Sigma‐Aldrich) at 37°C for 15 min and washed in water. Xylene was used to remove paraffin, as in Liu et al. (2011). Samples were viewed using a Nikon Eclipse E600 microscope (Nikon, Tokyo, Japan), and images were collected.

### 2.5 Determination of starch, sucrose, total carbon and nitrogen contents

Degraded and normal tubers, of similar size from the same seedlings, were sampled; they were sliced and their dry weight measured, as detailed above. A 30‐mg dried sample was used to measure sucrose and starch contents, as described by Fan et al. (2021). Starch content was determined using a starch kit (Megazyme International Ireland Ltd., Wicklow, Ireland). Total C and N concentrations in each sample were measured using an element analyzer (Vario El Cub; Elementar, Langenselbold, Germany), as in Zheng et al. (2018).

### 2.6 RNA extraction and quality control

The samples at 55 DAE from control in 2019 were used for transcriptomic analysis. Six independent samples of degradating and normal tubers of similar size were collected and stored in liquid N immediately. Total RNA was extracted using a Spectrum™ Plant Total RNA Kit (Sigma‐Aldrich), and its purity was checked using a NanoPhotometer® spectrophotometer (IMPLEN, CA, USA). RNA integrity was assessed using the RNA Nano 6000 Assay Kit of the Bioanalyzer 2100 system (Agilent Technologies, Santa Clara, CA, USA). The RNA concentration was measured using a Qubit® RNA Assay Kit and Qubit®2.0 Fluorometer (Life Technologies, Carlsbad, CA, USA). Based on the above‐mentioned parameters, RNA with an A_260_–A_280_ ratio value greater than 1.9 was selected for subsequent qRT‐PCR and sequencing analyses.

### 2.7 Library preparation and transcriptome sequencing

A total amount of 3 μg RNA per sample was used as input for building sequencing libraries with the NEBNext® Ultra™ RNALibrary Prep Kit for Illumina® (NEB, Ipswich, MA, USA), as described in an earlier study (Wang et al., 2017). Briefly, mRNA was purified from total RNA using poly‐T oligo‐attached magneticbeads. Fragmentation was performed using divalent cations under elevated temperature in NEBNext First Strand Synthesis Reaction Buffer (5×). First‐strand cDNA was synthesized using a random hexamer primer and M‐MuLV Leading Edge Genomic Services and Solutions Reverse Transcriptase (RNase H^−^). Second‐strand cDNA synthesis was subsequently performed using DNA polymerase I and RNase H. Remaining overhangs were converted into blunt ends via exonuclease/polymerase activities. After adenylation of 3′ ends of DNA fragments, NEBNext Adaptor with a hairpin loop structure was ligated to prepare for hybridisation. cDNA fragments 250 ~ 300 bp in length were preferentially selected and purified using the AMPure XP system (Beckman Coulter, Brea, CA, USA). Then, 3 μl USER enzyme mix (NEB) was applied with size‐selected, adaptor‐ligated cDNA at 37°C for 15 min, followed by 5 min at 95°C prior to PCR. PCR was performed using Phusion High‐Fidelity DNA polymerase, Universal PCR primers and Index (X) Primer. Finally, PCR products were purified using the AMPure XP system, and library quality was assessed using the Agilent Bioanalyzer 2100 system.

Clustering of the index‐coded samples was performed on a cBot Cluster Generation System using the TruSeq PE Cluster Kit v3‐cBot‐HS (Illumina, San Diego, CA, USA) in accordance with the manufacturer's instructions. After cluster generation, the library preparations were sequenced on an Illumina Hiseq platform, and 125/150 bp paired‐end reads were generated.

Reference genome and gene model annotation files were downloaded from genome website directly (https://www.ncbi.nlm.nih.gov/genome/?term=Solanum+tuberosum+L); the assembly and annotation information was Assembly SolTub_3.0 (accession GCF_000226075.1, annotation release ID: 101). Index of the reference genome was built using Hisat2 v2.0.5, and paired‐end clean reads were aligned to the reference genome using Hisat2 v2.0.5

### 2.8 GO enrichment analysis of differentially expressed genes

Analysis of differentially expressed genes (DEGs) under the two treatments was performed using the DESeq2 R package. The resulting *P* values were adjusted using Benjamini and Hochberg's approach for controlling the false discovery rate. Genes with an adjusted *P* value <0.05, as revealed by DESeq2, were classified as differentially expressed.

DEGs were also employed for the GO enrichment analysis implemented using the cluster Profiler R package, in which gene length bias was corrected (Young et al., 2010). The adjusted *P* value for significant GO terms was <0.05.

### 2.9 Quantitative real‐time PCR analyses

The Spectrum™ Plant Total RNA Kit (Sigma‐Aldrich) was used for total RNA extraction from potato tuber. After reverse transcription using the SuperScript RT‐PCR system (Invitrogen, Waltham, MA, USA), cDNA was used to perform the quantitative real‐time PCR analysis. IQ SYBR Green Supermix (Bio‐Rad, Hercules, CA, USA) and iCycle (Bio‐Rad) were used in our experiment. LOC102577640 was used as an internal standard for data normalization and quantification (Stritzler et al., 2017). The six genes selected for validating the expression profiles obtained from microarray hybridisations, and their primers, are listed in Table S1.

### 2.10 Statistical analyses

The data were analyzed using the SPSS statistical analysis package (version 19.0; SPSS Inc., Chicago, IL, USA). Means were tested by the least significant difference test at the *P* = 0.05 level.

## 3 RESULTS

### 3.1 Changes in tuber number during the growth period

**FIGURE 1:**
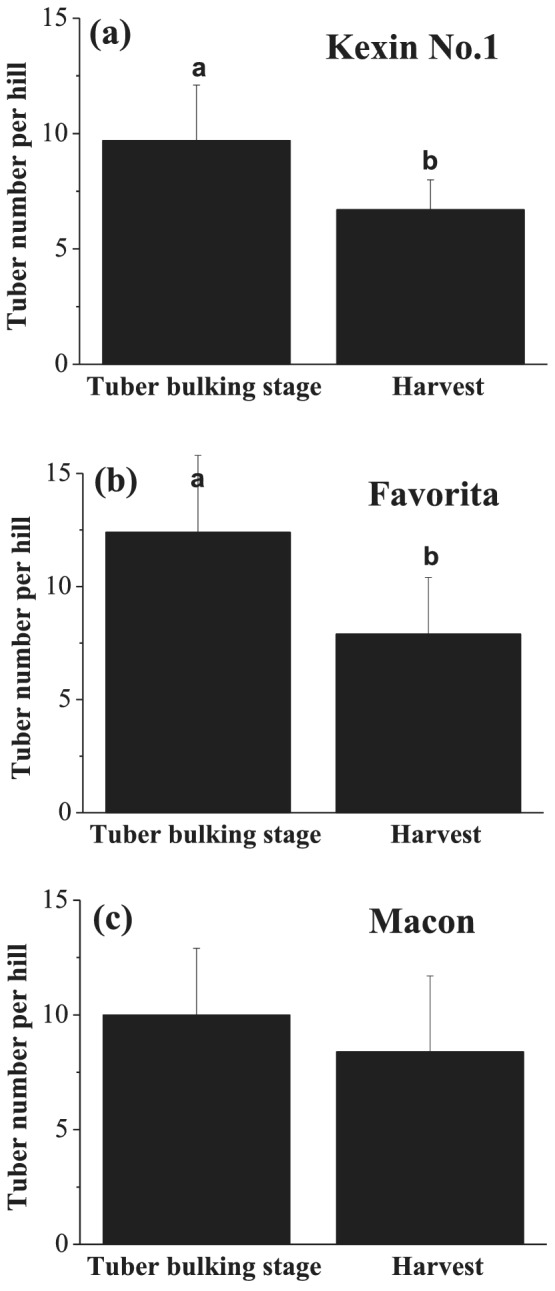
Tuber numbers per hill at the tuber bulking and harvesting stages for potato cultivars (a) Kexin No. 1, (b) Favorita, and (c) Macon in 2017. The number of tubers with a diameter >0.5 cm was calculated. The sampling dates were July 20 and August 20 (tuber bulking and harvest stages, respectively). The data are means and standard errors of 10 independent measurements. Different letters denote statistically significant differences at *P* <  0.05

Under normal growth conditions, the tuber numbers of three local potato cultivars—Kexin No. 1, Favorita, and Macon—were investigated in 2017. The data showed that total tuber numbers decreased at harvest (August 20) compared to the tuber bulking stage (July 20), in which cultivars Kexin No. 1 and Favorita exhibited a significant difference (Figure 1).

**FIGURE 2:**
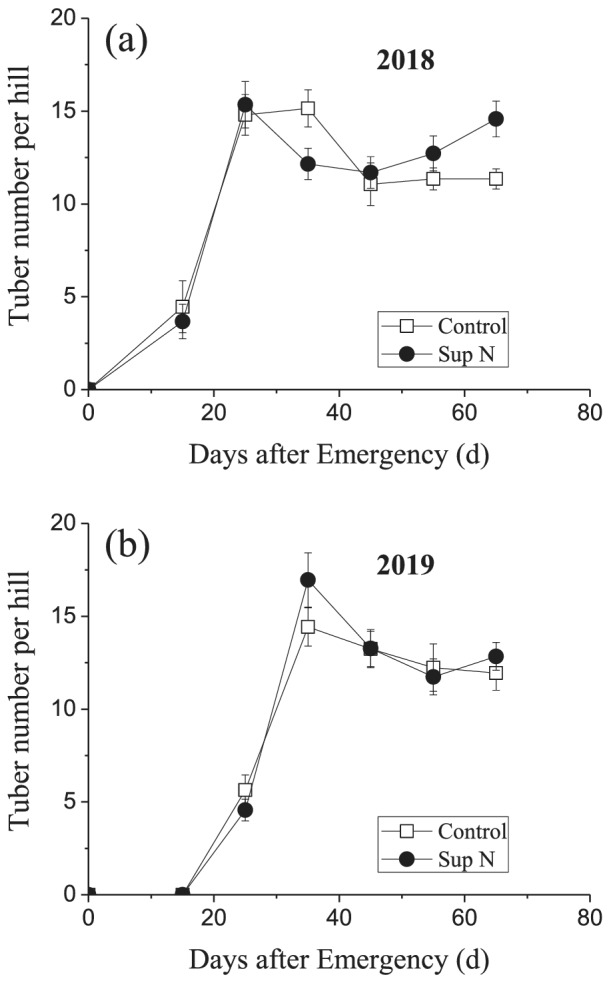
Changes in potato tuber numbers per hill during the entire developmental period, with (Sup N) and without (Control) N top‐dressing in (a) 2018 and (b) 2019. The cultivar Favorita was used as the experimental material, and all tubers were counted. Data are means and standard errors of 30 independent measurements

To validate and understand the changes in tuber numbers, the tuber numbers of the cultivar Favorita were investigated across the entire growth period in 2018 and 2019. The tuber numbers increased gradually, reaching a maximum at 35 DAE, and then decreased with or without N top‐dressing. The total number decreased gradually until harvest, when no N top‐dressing was applied, but exhibited a clear increase as some N was supplemented at a later developmental stage (Figure 2).

### 3.2 Tuber degradation and phenotype observation

**FIGURE 3:**
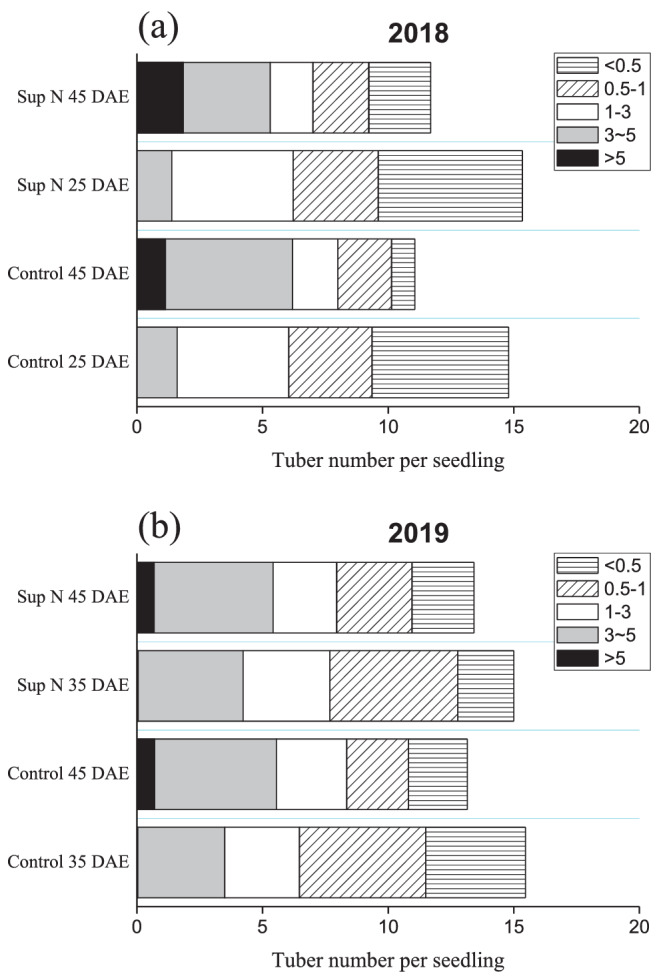
Numbers of tubers per hill at 25 and 45 DAE under N top‐dressing (Sup N) treatment in (a) 2018 and (b) 2019. Tuber diameter was classified as follows: <0.5, 0.5–1, 1–3, 3–5, and >5 cm. Data are means and standard errors of 30 independent measurements

To better understand why the tubers disappeared during development, we carried out a detailed investigation. Tuber size was classified based on the maximum swelling, as follows: <0.5 (tuber initial), 0.5–1, 1–3, 3–5, and >5 cm. Changes in tuber number by size was analyzed at 25 and 45 DAE in 2018; 4.51, 1.19, and 2.64 tubers decreased in size to <0.5, 0.5–1, and 1–3 cm, while 3.47 and 1.13 tubers increased in size to 3–5 and >5 cm under the control conditions, respectively; 3.28, 1.16, and 3.13 tubers decreased in size to <0.5, 0.5–1, and 1–3 cm, while 2.07 and 1.85 tubers increased in size to 3–5 and >5 cm under Sup N treatment, respectively (Figure 3a). Similar trends were found in 2019 when we compared the tuber numbers at 35 DAE (maximum) and 45 DAE (Figure 3b). The results suggested that 3 cm was a critical tuber diameter; tubers tended to increase above, or decrease below, 3 cm.

**FIGURE 4:**
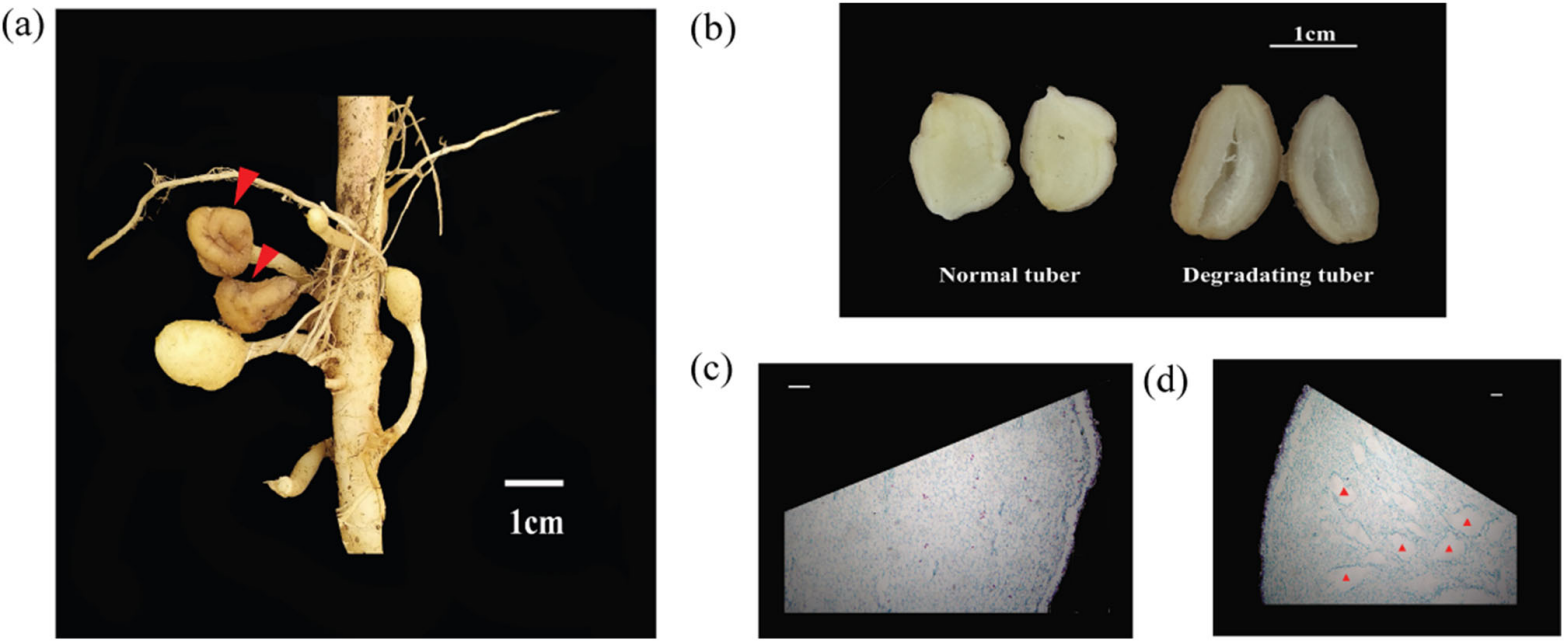
Phenotype of degradating tuber. (a) In situ observation of normal and degradating tubers (indicated by arrows) on a single seedling. (b) Hollow heart of degradating tubers was revealed by cutting with a knife along the midline of the stolon joint. (c) Cytological observation of a degradating tuber by paraffin sectioning

To determine whether the tubers that decreased in size or disappeared were smaller than 3 cm in diameter, phenotypic investigation was performed of the potato cultivar Favorita; it revealed that some tubers had brown or dark yellow skin and were shrinking or even becoming putrid, although many other tubers of similar size developed well in a single plant (Figure 4a). This was mainly observed on tubers around 1 cm in size; no tubers larger than 2 cm were observed to degrade. When the brown and shrinking tubers were cut in half, a hollow heart was revealed (Figure 4b). Paraffin sectioning showed that many cavities formed in degradating tubers, due to reduced amounts of pith and cortical cells (Figure 4c).

### 3.3 Tuber degradation is regulated by the source–sink relationship

**FIGURE 5:**
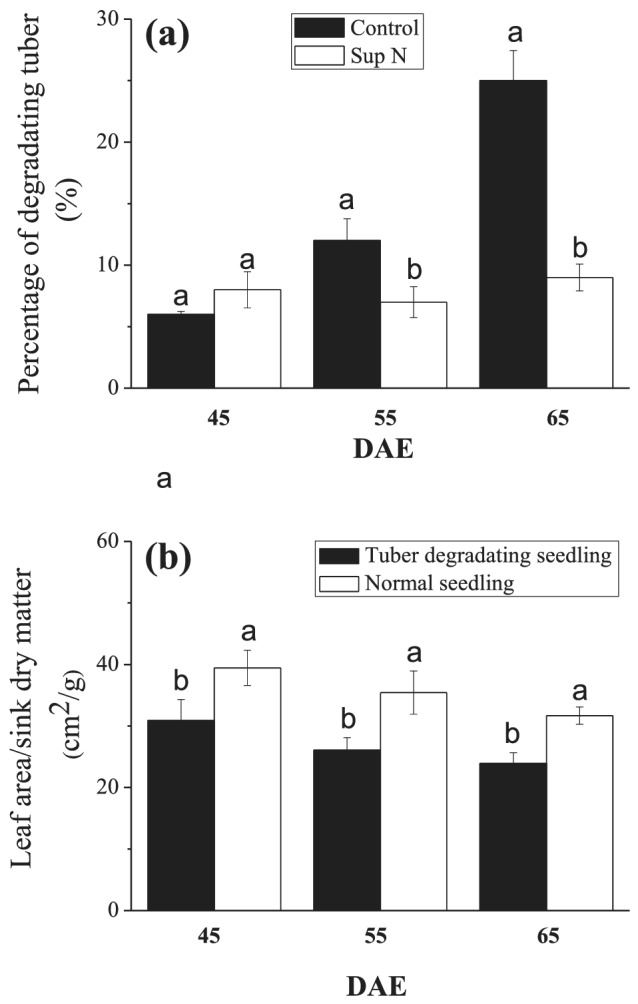
Source–sink relationship regulated by N supplementation: role in tuber degradation. (a) The percentage of degradating tubers relative to the total tuber number; the results were from the field trial in 2019; data are means and standard errors of 10 independent measurements. (b) The ratio of leaf area and tuber dry matter for normal and tuber‐degradating seedlings over the development period; the results were from the control in 2019; the data are means and standard errors of 30 independent measurements. Different letters denote statistically significant differences at *P* <  0.05

Tuber degradation was observed throughout the entire developmental period in 2019 field trial, from tuber initiation. However, the percentage of degradating tubers decreased significantly when N was supplied as a top‐dressing compared to the control (Figure 5a). To investigate whether tuber degradation was influenced by the source–sink relationship, the ratio of leaf area and tuber dry weight were used to express the source–sink relationship. Normal seedlings (without degradating tubers) and tuber‐degradating seedlings were selected for measurement and comparison. The results indicated that the tuber‐degradating seedlings had a lower ratio of leaf area and tuber dry weight (Figure 5b).

The contents of non‐structural carbohydrates, including starch and sucrose, were determined in both normal and degradating tubers and were higher in the former, particularly the starch contents (Table 1). The total C and N contents were also determined and were lower in degradating than normal tubers. The C:N ratio was consistent with the trends in C and N; that is, there was a sharper decline in C than N in the degradating tuber (Table 1).

**TABLE 1:** Comparison of carbon and nitrogen contents between normal and degradating tubers

Year	Sample	Starch (mg/g)	Sucrose (mg/g)	Carbon (mg/g)	Nitrogen (mg/g)	C/N
2018	Degradating tuber	67.16 ± 5.59 b	68.40 ± 13.21 a	364.37 ± 2.66 b	9.87 ± 1.21 b	25.19 ± 2.03 b
	Normal tuber	42.05 ± 25.72 a	71.25 ± 8.95 a	397.68 ± 3.51 a	12.54 ± .79 a	29.69 ± 2.83 a
2019	Degradating tuber	100.34 ± 15.73 b	54.32 ± 7.25 b	372.28 ± 5.39 b	10.45 ± .89 b	27.59 ± 2.43 b
	Normal tuber	300.84 ± 32.17 a	70.18 ± 12.53 a	405.57 ± 2.98 a	13.09 ± 1.04 a	32.09 ± 2.61 a

*Note*: Values within a column with the same letter are not statistically significantly different at *P* <  0.05.

### 3.4 Transcriptome analysis of the degradating tuber

RNA‐sequencing (RNA‐seq) of six libraries from the normal and degradating tubers (three replicates each) resulted in 314.26 million reads, of which more than 97% exhibited a quality score of Q20 (Table S2). Using Hisat2 as the mapping tool, 304.05 million high‐quality clean reads were aligned to 147,573 unigenes in the reference genome. The distribution of fragments per kilobase of transcript per million mapped reads (FPKM) values was used to investigate the gene expression pattern in each sample. Correlations of the expression patterns across normal and degradating tuber samples are presented in Figure S1. The correlation coefficients for the three replicates were all greater than 0.85. The results of principal component analysis (PCA) of all genes detected in the six libraries were plotted and confirmed the similarity of the transcriptomes among samples from normal and degradating tubers, as illustrated in Figure S2.

The DEGs between normal and degradating tubers were analyzed based on the transcriptional profiles. There were 514 and 417 up‐regulated and down‐regulated genes in the analysis of normal tubers (EdgeRpadj <  0.05, |log2FoldChange| > 1.0). The top 10 up‐regulated and down‐regulated genes are detailed in Table 2. The top 10 up‐regulated genes included two carbohydrate‐related genes: a polysaccharide degradation‐related gene LOC102601831 and a sugar transport gene LOC102587850 (SWEET6a). The other eight genes were 8‐hydroxygeraniol dehydrogenase, calcium uniporter protein, indole‐3‐acetic acid‐amido synthetase, endonuclease, and four genes of unknown function. The top 10 down‐regulated genes included 6 proteinase inhibitor genes; the remaining 4 included cytochrome P450, patatin‐like phospholipase, and 2 probable lipoxygenase genes (Table 2).

**TABLE 2:** Top 10 up‐regulated and down‐regulated DEGs in degradating tubers

Gene ID	Log2Fold	P‐value	Padj	Gene name	Gene description
	Change				
**Up‐regulated genes**					
102600145	13.97	1.02E‐07	2.16E‐04	LOC102600145	8‐hydroxygeraniol dehydrogenase‐like
102601831	13.39	8.71E‐07	6.84E‐04	LOC102601831	Polygalacturonase‐like
102580870	11.09	6.85E‐07	6.69E‐04	LOC102580870	Miraculin
107063347	11.08	3.27E‐05	4.69E‐03	LOC107063347	Putative uncharacterised protein DDB_G0277255
102593741	10.39	2.31E‐05	3.82E‐03	LOC102593741	Coiled‐coil domain‐containing protein 15‐like
102597758	10.25	1.03E‐06	6.93E‐04	LOC102597758	Miraculin‐like
102587850	10.05	8.27E‐05	7.74E‐04	LOC102587850	Bidirectional sugar transporter SWEET6a
102580387	9.89	8.38E‐04	3.23E‐02	LOC102580387	Calcium uniporter protein 2, mitochondrial‐like
102580803	9.79	1.30E‐06	7.59E‐04	LOC102580803	Indole‐3‐acetic acid‐amido synthetase GH3.6‐like
102603473	9.63	1.00E‐04	8.72E‐03	LOC102603473	Endonuclease 2
**Down‐regulated genes**					
102595246	−13.25	1.21E‐06	7.20E‐04	LOC102595246	Cysteine protease inhibitor 8‐like
102604582	−13.09	6.24E‐06	1.77E‐03	LOC102604582	Aspartic protease inhibitor 3
102589278	−12.31	5.58E‐07	6.13E‐04	LOC102589278	Latex serine proteinase inhibitor‐like
102595151	−12.3	4.04E‐10	5.55E‐06	LOC102595151	Cytochrome P450 82C4‐like
102597545	−12.29	5.07E‐05	6.16E‐03	LOC102597545	Chymotrypsin inhibitor I, A, B and C subunits‐like
102577615	−12.23	3.07E‐09	1.69E‐05	LOC102577615	5‐lipoxygenase
102580584	−12.19	3.21E‐06	1.29E‐03	LOC102580584	Serine protease inhibitor 1
Novel.1459	−12.19	2.92E‐06	1.22E‐03	‐	PF01734:Patatin‐like phospholipase
102598541	−11.59	1.12E‐06	7.16E‐04	LOC102598541	Probable linoleate 9S‐lipoxygenase 8‐like
102577839	−11.53	1.32E‐05	2.72E‐03	LOC102577839	Proteinase inhibitor

### 3.5 GO analysis of DEGs

**FIGURE 6:**
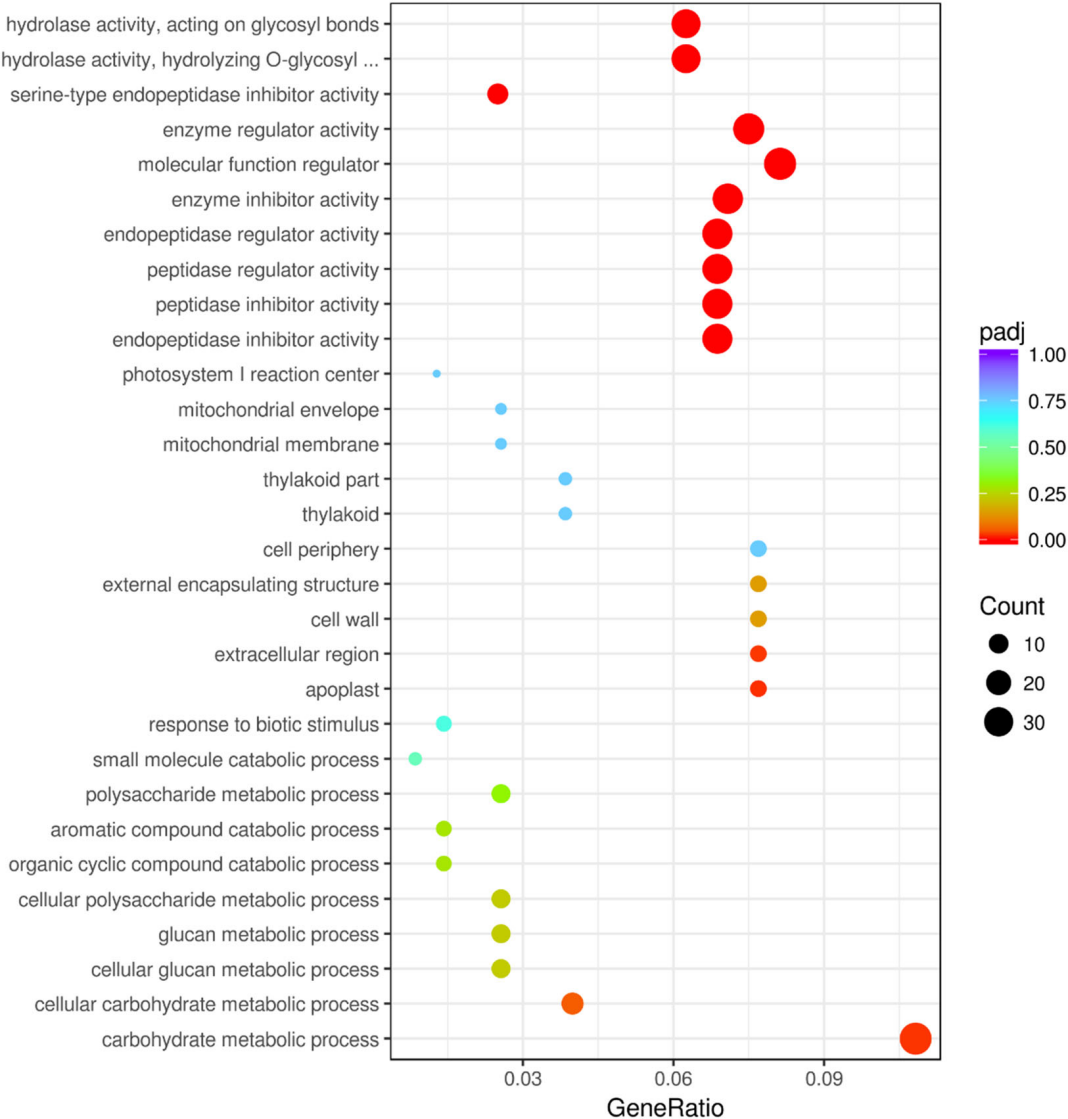
GO enrichment analysis of DEGs in normal and degradating tubers. The *x* axis indicates the proportion of annotated DEGs relative to all DEGs, and the *y* axis shows the top 10 GO terms including MF, CC, and BP. The size of the dot represents the number of annotated GO terms, and the color of the dot represents the padj value

GO enrichment analysis was used to identify DEGs in the degradating tubers. The DEGs were classified into three categories: biological processes (BP), molecular functions (MF), and cellular components (CC). A total of 351 DEGs (175 down‐regulated and 176 up‐regulated) were used for BP ontology (Table S3). The dominant five BP terms were “carbohydrate metabolic process,” “cellular carbohydrate metabolic process,” “cellular glucan metabolic process,” “glucan metabolic process,” and “cellular polysaccharide metabolic process,” all of which are involved in carbohydrate metabolism‐related processes (Figure 6). In total, 480 DEGs (239 down‐regulated and 241 up‐regulated) were involved in MF ontology (Table S3). The dominant GO terms were “peptidase inhibitor activity,” “peptidase regulator activity,” “enzyme inhibitor activity,” “molecular function regulator,” “enzyme regulator activity,” and “hydrolase activity” (Figure 6). There were 78 DEGs involved in CC ontology, of which 38 were down‐regulated and 40 were up‐regulated in degradating tubers. The dominant terms were “apoplast,” “extracellular region,” “cell wall,” “external encapsulating structure,” and “cell periphery” (Figure 6).

### 3.6 RNA‐seq validation by qRT‐PCR

**FIGURE 7:**
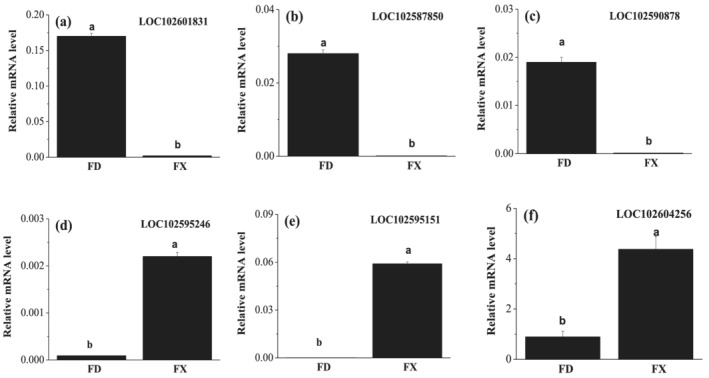
Verification of the relative expression levels of DEGs by qRT‐PCR. The selected genes are (a) LOC102601831, (b) LOC102587850, (c) LOC102590878, (d) LOC102595246, (e) LOC102595151, and (f) LOC102604256. FX and FD in figures represent normal and degradating tubers, respectively. All qRT‐PCR analyses involved three biological replicates. Error bars represent standard deviation

To confirm the RNA‐seq data, six genes showing differential expression between normal and degradating tubers were randomly selected for qRT‐PCR analysis. The three up‐regulated genes are all related to carbohydrate metabolism. Another three down‐regulated genes are related to peptidase inhibitor activity. The selected genes are not only used for RNA‐seq data confirmation but also helpful for understanding the biological process of tuber degradating. The expression tendencies of these genes agreed well with the RNA‐seq results, indicating good reliability of the RNA‐seq results (Figure 7).

## 4 DISCUSSION

Tuber number is an essential factor determining yield and commodity in potato production. A suitable number of tubers having a uniform size distribution is expected in commercial potatoes, but the number should be maximized for seed potato production. Therefore, the patterns and mechanisms of tuber number is an important research target. Extensive research has focused on tuberization in recent decades (Navarro et al., 2011; Zierer et al., 2021), but less attention has been paid to subsequent developmental changes in tubers. We found that not all initiated tubers swelled continuously until harvest. The total tuber numbers declined compared to the maximum at the end of the growth season (Figure 2). Our investigation of three different cultivars revealed that this phenomenon is widespread (Figure 1).

Further in situ observations in the field revealed that some tubers exhibited brown or dark yellow skin, shrinkage and hollow hearts, and even decomposition in some normal‐growth seedlings (Figure 4a,b). Moreover, these events occurred throughout the entire potato development period. The decrease in tuber number was the result of tuber shrinkage and decomposition, which ultimately led to tuber disappearance. Degradation was observed only in tubers less than 3 cm in diameter (Figure 3), mainly in those around 1 cm in diameter according to the field observations.

Organ abscission is crucial for the natural development of plant parts, such as leaves, flowers, and fruits (Aloni et al., 1997; Gómez‐Cadenas et al., 2000; Reichardt et al., 2020; Zhu et al., 2011). Many studies have observed that organ abscission is often induced by competition for carbohydrates, reflected in “early flower drop” in many plants (Gómez‐Cadenas et al., 2000; Liang et al., 2020). Abscission is not always negative: The “June drop” seen in fruit tree cultivars can help apple growers avoid the negative impact of excessive fruit bearing (McFadyen et al., 2011). This suggests that tuber degradation is closely correlated with competition for carbohydrates.

In this study, the carbohydrate contents of degradating tubers were significantly lower compared to normal tubers (Table 1). GO analysis of transcriptomes revealed that the top 5 BP affected by tuber degradation were all related to carbohydrate metabolism (Figure 6). DEG analyses revealed that two carbohydrate‐related genes were dramatically up‐regulated, including a polysaccharide degradation‐related gene (LOC102601831). Increased expression of this gene may be related to carbohydrate remobilization. Because polysaccharides must be broken down before they can be remobilized and transported to other regions (Stitt & Zeeman, 2012; Wang et al., 2020), carbohydrate transport is thought to play a critical role in regulating abscission, as demonstrated in other plants (Jones et al., 2016; Liang et al., 2020). Another markedly up‐regulated gene in degradating tubers was the sugar transport gene, LOC102587850 (SWEET6a) (Table 2). As mentioned above, SWEET family proteins are key for sucrose efflux (Chen et al., 2012; Jeena et al., 2019). Organ abscission activated by sucrose deprivation has been shown to play a role in flower dropping (Liang et al., 2020); tuber degradation may occur via the same mechanism.

Tuber degradation is a senescence‐ and death‐related process at the cellular level. Several hydrolytic enzymes are involved in programmed cell death, including various proteases and nucleases (Hautegem et al., 2015; Thomas, 2013). The MF showing the most changes according to our GO analysis were “peptidase inhibitor activity” and “hydrolase activity” (Figure 6). Of the top 10 down‐regulated DEGs, 6 were proteinase inhibitor genes, including cysteine protease inhibitor 8‐like, aspartic protease inhibitor 3, and serine protease inhibitor 1 (Table 2). Under the actions of these enzymes, cells collapse and matter remobilizes, forming aerenchyma‐like structures (such as phosphorus‐stressed maize roots in degradating tubers) (Fan et al., 2003).

Supplementing with N during tuber development attenuates tuber degradation (Figures 2 and 5). Previous study showed that the N supply delaying tuber formation might be via reducing the C:N ratio in seedling (Krauss & Marschner, 1982; Sarkar & Naik, 1998; Zheng et al., 2018). The C:N ratio is also reduced in degradating tuber which means more C‐contained than N‐contained products efflux. N is a determinant of plant photosynthate, altering carbohydrate metabolism and sugar signaling at source (Hörtensteiner & Feller, 2002; Thomas, 2013). Additional N supplement might have little influence on the C:N ratio in degradating tuber, which is different from the regulatory mechanism of C:N ratio in tuberization. Sufficient N supply mainly promotes greater carbohydrate accumulation, which better meets the sink tuber demand for nutrition; a balanced source/sink relationship alleviates sink tuber competition (Figure 5b).

In conclusion, the abscission of flowers, fruits, and leaves means that tuber degradation is crucial for potato development. The source–sink relationship and carbohydrate metabolism play crucial roles in tuber degradation. Suitable N management strategies in accordance with the aims of potato production may regulate final tuber numbers by modulating their degradation.

## AUTHOR CONTRIBUTIONS

M.F., L.J., and K.H. designed the research; K.H., Q.S., and Y. Q performed the research; J.Y. and K.L. carried out data analysis: L.J. wrote the article; M. F revised the article.

## CONFLICT OF INTEREST

The Authors did not report any conflict of interest.

## Supporting information


**Table S1.** Primers used for qRT‐PCR.
**Table S2.** Results of raw read filtering. FX1–FX3 are normal tuber samples; FD1–FD3 are degradating tuber samples.
**Table S3.** GO enrichment analysis of degradating tubers. BP: biological processes, MF: molecular functions, CC: cellular components.

**Figure S1.** Pearson correlation analysis of gene expression patterns among samples.
**Figure S2.** PCA of all expressed genes.
**Figure S3.** In situ observation of normal and degradating tubers on a single seedling.

**Data S1.** Supporting Information

## Data Availability

RNA‐sequencing data have been deposited with the NCBI (Accession Number: PRJNA777000).
