# Supplementary material for: Potato tuber degradation is regulated by carbohydrate metabolism: Results of transcriptomic analysis
Source: Plant Direct. 2022 Jan 14;6(1):e379. doi: 10.1002/pld3.379 (PMC8758968; doi:10.1002/pld3.379)
Supplement: Supplementary file 1 — Table S1. Primers used for qRT‐PCR. Table S2. Results of raw read filtering. FX1–FX3 are normal tuber samples; FD1–FD3 are degradating tuber samples. Table S3. GO enrichment analysis of degradating tubers. BP: biological processes, MF: molecular functions, CC: cellular components. [file PLD3-6-e379-s002.docx]

Table S1. Primers used for qRT-PCR determination

| Gene_name | Forward primer | Reverse primer |
| --- | --- | --- |
| LOC102577640 | ATTGGAAACGGATATGCTCCA | TCCTTACCTGAACGCCTGTCA |
| LOC102601831 | TGACTCCACCATTGGCACAGG | GCTTCTGAATTTCCACCTCCCA |
| LOC102587850 | ATCCCTACATCTTGACTGGAAACG | TCCACTTTAGATGGCTTCTCGTC |
| LOC102590878 | ACAATACACGGAAATGGGACTCA | GGACCACGATAATCACACGGAT |
| LOC102595246 | ATTGAGAAATATGAGACAGGGAGACC | GCTTGTTATTGAGAGCCAGACGG |
| LOC102595151 | TTTGAGTTCATCCCGTTTGGTT | CACAGGTTTTGCTTTAGGCATAGT |
| LOC102604256 | GCCCTATTACTCCCCCTTTTCT | TTGACAAGAGCCAAACGCCT |

Table S2. The results of raw reads filtering

| Samples | Total raw reads | Total clean reads | Q20 (%) | GC(%) |
| --- | --- | --- | --- | --- |
| FX1 | 47644458 | 46325850(97.23%) | 97.54 | 42.58 |
| FX2 | 46752250 | 45315474(96.93%) | 97.41 | 42.14 |
| FX3 | 50069638 | 48322712(96.51%) | 97.57 | 42.65 |
| FD1 | 60643728 | 58410714(96.32%) | 97.34 | 41.65 |
| FD2 | 58639228 | 56907498(97.05%) | 97.37 | 41.58 |
| FD3 | 50507536 | 48771664(96.56%) | 97.19 | 41.94 |

Note: FX1, FX2, FX3 are normal tuber samples; FD1, FD2, FD3 are degradating tuber samples.
