# Supplementary material for: Potato tuber degradation is regulated by carbohydrate metabolism: Results of transcriptomic analysis
Source: Plant Direct. 2022 Jan 14;6(1):e379. doi: 10.1002/pld3.379 (PMC8758968; doi:10.1002/pld3.379)
Supplement: Supplementary file 2 — Figure S1. Pearson correlation analysis of gene expression patterns among samples. Figure S2. PCA of all expressed genes. Figure S3. In situ observation of normal and degradating tubers on a single seedling. [file PLD3-6-e379-s001.docx]

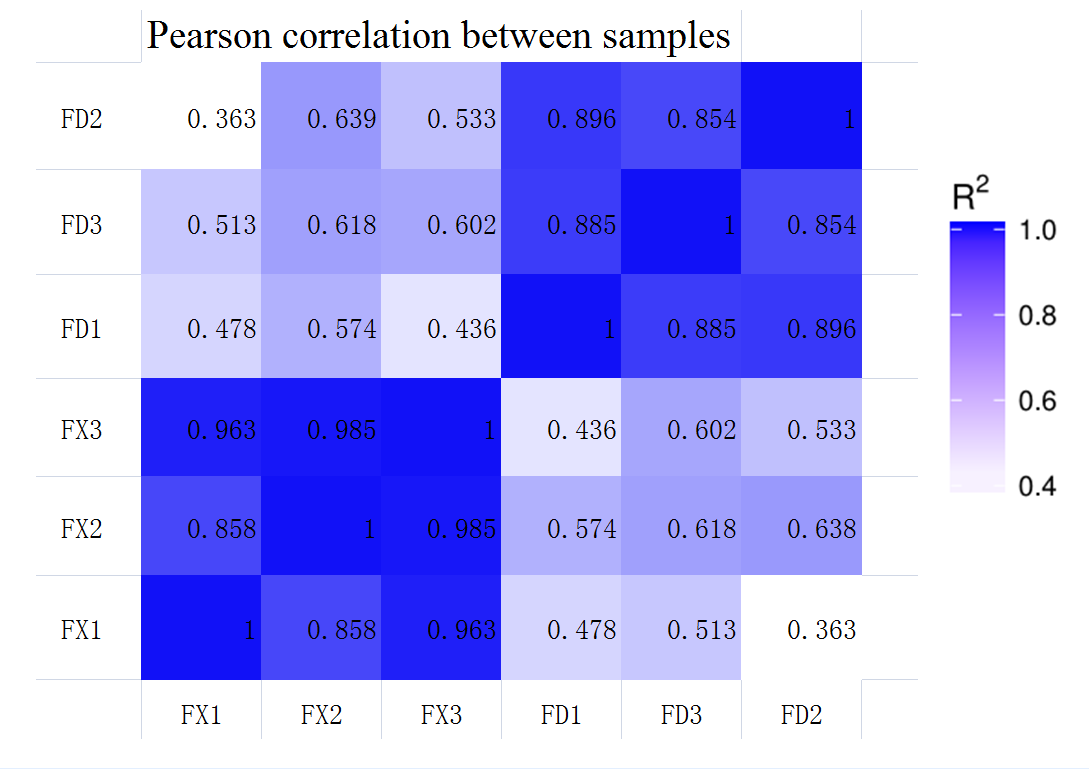


**Supplementary Fig. S1** The correlation of gene expression pattern as Pearson’s correlations among the samples. The number in the box approximal to 1 means the similarity degree is high. The blue color represents the high correlation, the white color refers to the low correlation.


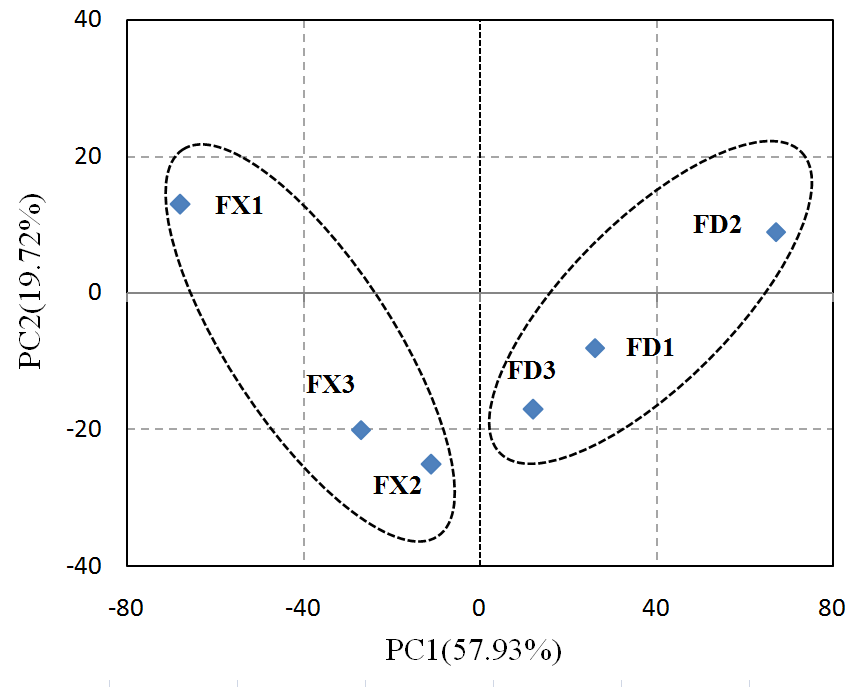


**Supplementary Fig. S2** Principal component analysis (PCA) based on all the expressed genes. Two distinct groups are clustered based on the gene expression level of each sample.


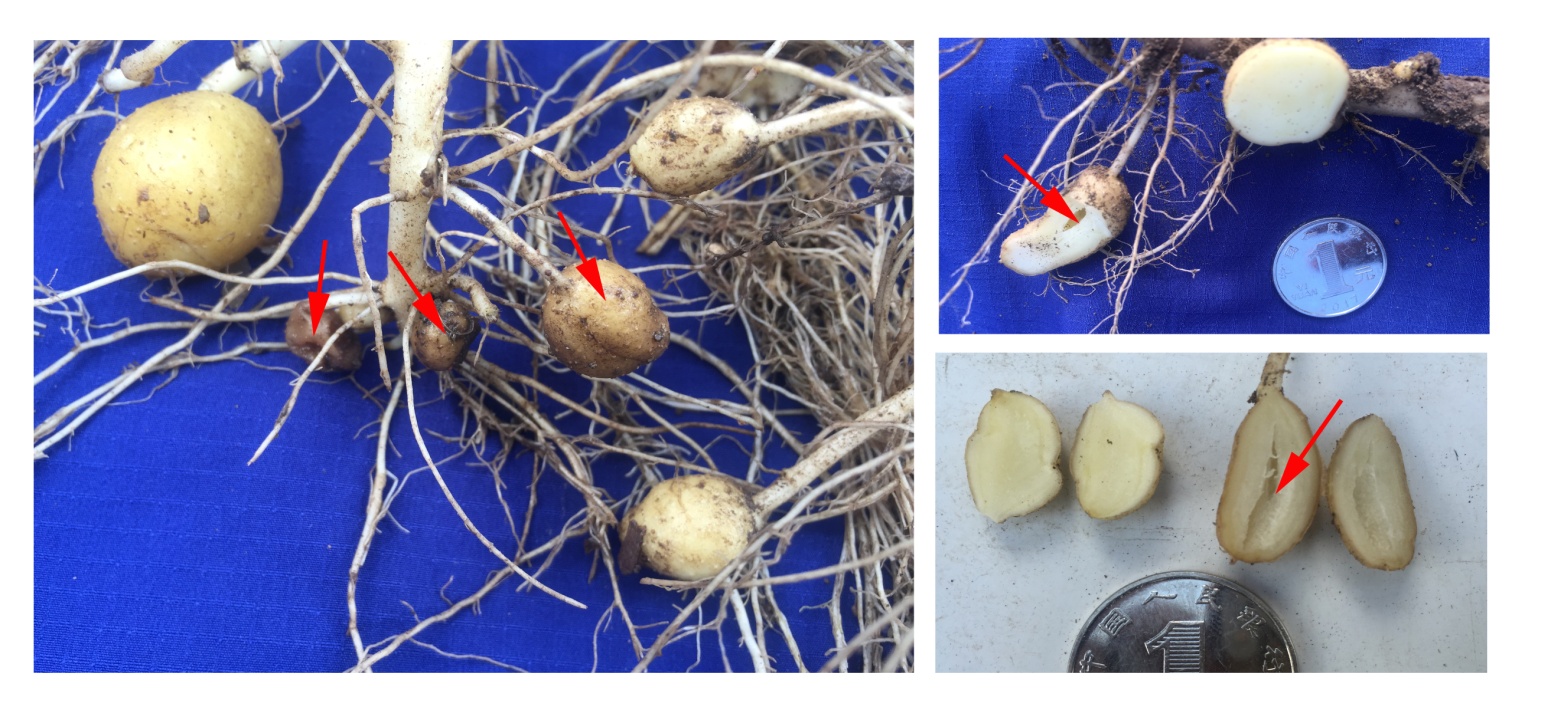


**Supplementary Fig. S3** *In situ* observation of normal and degradating tubers (indicated by arrows) on a single seedling. The seedling in the photo was Favorita cultivar at the 65 DAE in 2018 taken in our experimental plot.
